# Beneficial islet inflammation in health depends on pericytic TLR/MyD88 signaling

**DOI:** 10.1172/JCI179335

**Published:** 2024-06-17

**Authors:** Anat Schonblum, Dunia Ali Naser, Shai Ovadia, Mohammed Egbaria, Shani Puyesky, Alona Epshtein, Tomer Wald, Sophia Mercado-Medrez, Ruth Ashery-Padan, Limor Landsman

**Affiliations:** 1 Department of Cell and Development Biology, Faculty of Medical and Health Sciences and; 2 Department of Human Molecular Genetics and Biochemistry, Faculty of Medical and Health Sciences and Sagol School of Neuroscience, Tel Aviv University, Tel Aviv, Israel.

**Keywords:** Inflammation, Metabolism, Diabetes, Islet cells, Pericytes

## Abstract

While inflammation is beneficial for insulin secretion during homeostasis, its transformation adversely affects β cells and contributes to diabetes. However, the regulation of islet inflammation for maintaining glucose homeostasis remains largely unknown. Here, we identified pericytes as pivotal regulators of islet immune and β cell function in health. Islets and pancreatic pericytes express various cytokines in healthy humans and mice. To interfere with the pericytic inflammatory response, we selectively inhibited the TLR/MyD88 pathway in these cells in transgenic mice. The loss of MyD88 impaired pericytic cytokine production. Furthermore, MyD88-deficient mice exhibited skewed islet inflammation with fewer cells, an impaired macrophage phenotype, and reduced IL-1β production. This aberrant pericyte-orchestrated islet inflammation was associated with β cell dedifferentiation and impaired glucose response. Additionally, we found that Cxcl1, a pericytic MyD88-dependent cytokine, promoted immune IL-1β production. Treatment with either Cxcl1 or IL-1β restored the mature β cell phenotype and glucose response in transgenic mice, suggesting a potential mechanism through which pericytes and immune cells regulate glucose homeostasis. Our study revealed pericyte-orchestrated islet inflammation as a crucial element in glucose regulation, implicating this process as a potential therapeutic target for diabetes.

## Abstract 2

Pericytes serve as regulators of inflammation, with supportive effects on β cells and glucose homeostasis.

## Graphical abstract

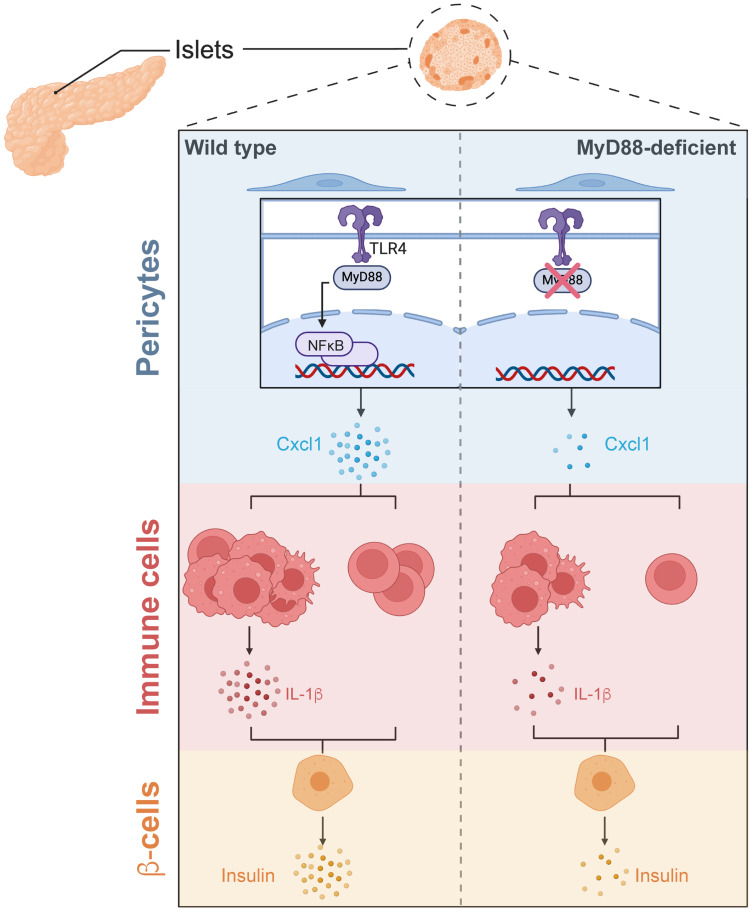

## Introduction

β Cell function is continuously regulated to facilitate proper insulin secretion and maintain glucose homeostasis. Disruption of this regulation leads to β cell dedifferentiation, characterized by the loss of their mature functional phenotype, ultimately contributing to the development of type 2 diabetes (T2D) (1–5). Recently, the islet microenvironment has been identified as a crucial regulator of β cell function in health and its impairment in diabetes (6, 7). However, the precise nature of this regulation is not well defined.

A growing body of evidence has established T2D as an inflammatory disease (8–11). Interestingly, while deleterious islet inflammation contributes to disease progression, recent studies have demonstrated a positive role for islet immune cells, primarily macrophages, in maintaining β cell mass and function (12–19). Islet inflammation has been proposed to promote β cell adaptation to increased metabolic stress, which is consistent with the increasingly recognized role of sterile inflammation (i.e., inflammation that is not triggered by an infectious agent) in tissue remodeling (20). This constructive islet inflammation relies on the tightly regulated activities of multiple cell populations, including macrophages, DCs, B cells, and T cells. When this tightly orchestrated inflammatory response fails, it transforms from a constructive to a destructive force, contributing to β cell dysfunction and T2D progression (8, 9, 21–25).

Macrophages, the predominant islet immune cell population, support β cell recovery after damage by secreting IGF1, CTGF, and other factors (7, 12, 15, 17, 26). Additionally, these cells promote insulin secretion by producing retinoic acid and interleukin 1β (IL-1β) (8, 14). Notably, IL-1β may also have a deleterious effect on β cells, likely depending on the context and duration of exposure (27, 28). Macrophages have also been suggested to contribute to β cell dysfunction and T2D progression by undergoing a phenotypic shift that alters the repertoire of secreted factors (8, 22, 24, 29, 30). During stress, monocyte recruitment to islets and macrophage activation are regulated by the islet vasculature (7, 12). However, the mechanism by which islet inflammation is regulated to allow proper β cell function remains unknown.

Pericytes are contractile cells that surround the endothelial cells of capillaries and, together with vascular smooth muscle cells, constitute a class of cells termed “mural cells” (31). The perivascular localization of pericytes positions them ideally to control various aspects of the immune response, including recruitment, extravasation, and activation (32). Pericytes have been shown to act as immunoregulators in various tissues, such as the brain and kidneys, where they secrete cytokines to facilitate a robust response to damage (32–38). Within pancreatic islets, pericytes directly regulate β cell function and their mature phenotype by secreting factors such as nerve growth factor (NGF), BMP4, and extracellular matrix components (39–43). Additionally, pericytes indirectly support glucose regulation by secreting IL-33, which promotes retinoic acid production by immune cells (14, 44). Changes in islet pericytes have been associated with impaired glucose regulation and diabetes (45–47). Despite these insights, the specific role of pancreatic pericytes in regulating islet inflammation has yet to be determined.

While the impact of islet inflammation on glucose homeostasis is recognized, how it is regulated remains unclear. Islets and pancreatic pericytes of healthy humans and mice express various cytokines. We hypothesized that pericytes regulate islet inflammation and that pericyte-orchestrated islet inflammation supports β cell function and glucose homeostasis. To test this hypothesis and intervene in the pericytic inflammatory response, we selectively inhibited MyD88, a canonical adaptor of the TLR/IL-1 receptor (IL-1R) pathway, in transgenic mice. Islets deficient in pericytic MyD88 displayed altered islet inflammation characterized by decreased cell numbers, abnormal macrophage phenotype, and lower IL-1β expression. This aberrant inflammatory response is associated with β cell dedifferentiation, inadequate insulin secretion, and an impaired glucose response. Focusing on Cxcl1, a pericytic TLR4/MyD88-dependent cytokine, we found that this cytokine maintains immune cell number and promotes the expression of IL-1β by these cells. Treatments with either Cxcl1 or IL-1β restored the mature β cell phenotype and rescued MyD88-deficient mice from glucose intolerance, indicating the Cxcl1/IL-1β axis as a potential therapeutic target. Our findings highlight the critical role of pericyte-orchestrated islet inflammation in the glucose regulatory network.

## Results

### Pancreatic pericytes of healthy humans and mice express cytokines.

**Figure 1:**
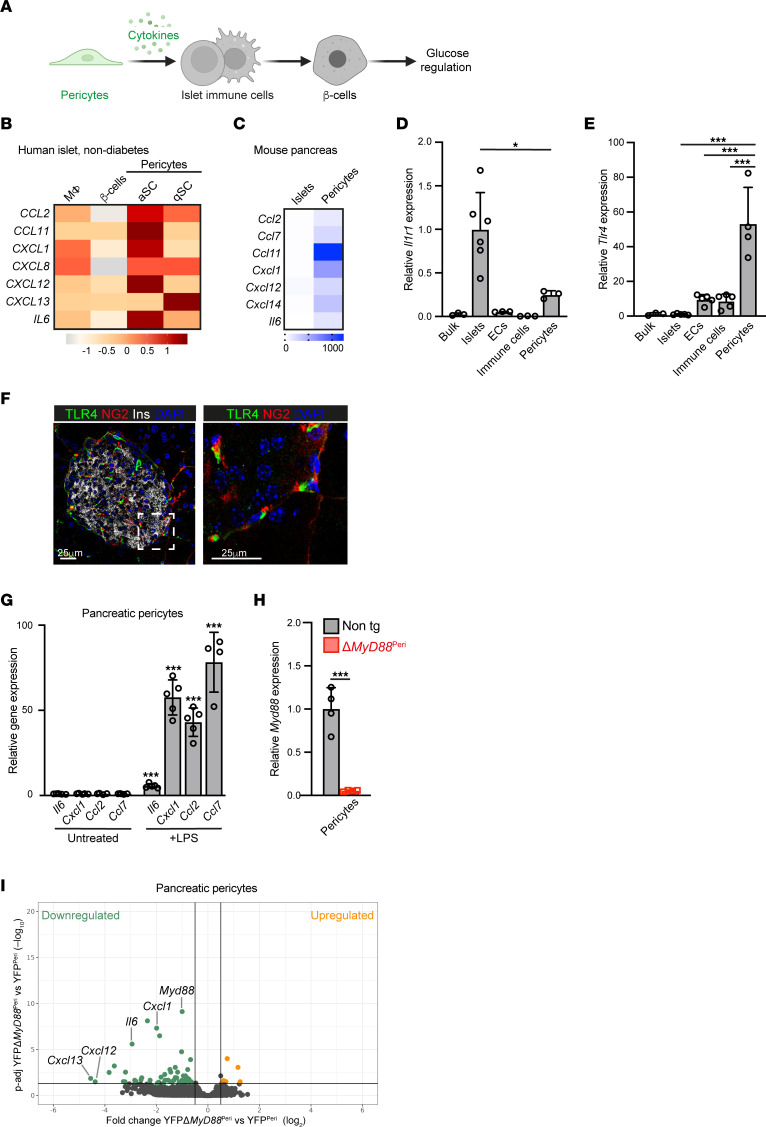
Pancreatic pericytes express cytokines in a TLR4/MyD88-dependent manner. (**A**) A graphical model of the study’s hypothesis, with pericytes highlighted in green. (**B**) Heatmap showing the relative expression of selected cytokines in macrophages, β cells, and pericytes (originally annotated as “quiescent stellate [qSC]” and “activated stellate [aSC]”), employing published scRNA-seq analysis of islets from healthy human donors (48). (**C**) Heatmap showing the relative expression of selected cytokines in isolated pericytes and islets, employing a previously published RNA-seq analysis of mouse pancreata (41). (**D** and **E**) Bar diagram (mean ± SD) showing the results of qPCR analysis of *Il1r1* (**D**) and *Tlr4* (**E**) transcripts in bulk mouse pancreatic tissues, isolated islets (average set to 1), pancreatic endothelial cells (ECs; PECAM1^+^), pancreatic immune cells (CD45^+^), and pancreatic pericytes (purified based on YFP expression from *Nkx3-2*-Cre;*R26*-YFP mice). *n* = 3–6. *P < 0.05, ***P < 0.005 (unpaired, 2-tailed Student’s *t* test). Each dot represents a single sample. (**F**) Immunofluorescence analysis of adult mouse pancreatic tissue sections for TLR4 (green), the pericytic marker NG2 (red), insulin (white), and DAPI (blue). The right panel shows a higher magnification of the area framed in the white box on the left panel. Scale bars: 25 μm. (**G**) Cultured neonatal pancreatic pericytes were either treated with LPS (right) or left untreated (left; the average was set to 1) and harvested after 24 hours. Bar diagrams (mean ± SD) showing the expression levels of genes encoding selected cytokines analyzed by qPCR. *n* = 5. One representative of 2 independent experiments. ***P < 0.005 (unpaired, 2-tailed Student’s *t* test) compared with the untreated group. Each dot represents a single sample. (**H**) Bar diagram (mean ± SD) showing the results of qPCR analysis of *Myd88* transcripts in pancreatic pericytes purified from *Nkx3-2*-Cre;*R26*-YFP (gray) or *Nkx3-2*-Cre;*Myd88^fl/fl^*;*R26*-YFP (Δ*MyD88*^Peri^; red) mice based on YFP expression. *n* = 4. ***P < 0.005 (unpaired, 2-tailed Student’s *t* test) compared with nontransgenic mice. Each dot represents a single sample. (**I**) RNA-seq analysis of pancreatic pericytes from control YFP^Peri^ (*Nkx3-2*-Cre;*R26*-YFP) and YFPΔ*MyD88*^Peri^ (*Nkx3-2*-Cre;*Myd88^fl/fl^*;*R26*-YFP) 15-week-old mice. Volcano plot analysis showing genes upregulated (orange) and downregulated (green) in YFPΔ*MyD88*^Peri^ pericytes. Selected genes are annotated. *n* = 3.

We hypothesized that islet inflammation, which is fundamental for β cell function and glucose regulation, is regulated by pericytes (Figure 1A). In many tissues, pericytes produce inflammatory mediators, including cytokines (32, 34–37). To determine whether this finding is valid for the pancreas, we used publicly available transcriptome analyses of human and mouse pancreatic cells (41, 48). To define cytokine expression by pericytes in human islets, we utilized single-cell RNA-seq (scRNA-seq) analysis of islets isolated from donors without diabetes or detectable autoantibodies (48). Human islet pericytes (identified by the expression of the pericytic markers *PDGFRB*, *RGS5*, and *ACTA2*, but not the stellate cell marker *GFAP*, originally annotated as “quiescent stellate” and “activated stellate”; Supplemental Figure 1; supplemental material available online with this article; https://doi.org/10.1172/JCI179335DS1) (46, 48) express cytokine-encoding genes, including *CCL2*, *CCL11*, *CXCL1*, *CXCL12*, *CXCL13*, and *IL6*, at higher levels than macrophages and β cells (Figure 1B). Next, we used our previously published RNA-seq analysis of pancreatic pericytes from healthy young mice (41). As shown in Figure 1C, mouse pancreatic pericytes expressed an array of genes encoding cytokines, some of which were also expressed by human islet pericytes (*Ccl2*, *Ccl11*, *Cxcl1*, *Cxcl12*, and *Il6*; Figure 1, B and C). Although some of these cytokines have been implicated in glucose regulation (14, 15, 49–54), their pancreatic sources remain largely unknown.

### The TLR/IL-1R pathway is active in pancreatic pericytes.

To study the role of pericytic cytokines, we interfered with their production. One of the main pathways regulating cytokine expression is the TLR/IL-1R pathway. Mouse pancreatic pericytes express 2 of the main receptors in this pathway, *Tlr4* and *Il1r1* (Supplemental Figure 1). A comparison of the expression of these 2 receptors in various pancreatic cell types revealed that pericytes (purified based on their fluorescent labeling in *Nkx3-2*-Cre;*R26*-YFP mice) (41, 42, 55) expressed lower levels of *Il1r1* than did endocrine cells, while they expressed *Tlr4* at significantly higher levels than did the other pancreatic cell populations (Figure 1, D and E). To verify the presence of pericytic TLR4 at the protein level and independently of Nkx3-2/YFP expression, we performed immunofluorescence analysis of a wild-type pancreas, which indicated the presence of this receptor in islet-associated pericytes (Figure 1F). Finally, to determine whether the TLR/IL-1R pathway is active in pancreatic pericytes, we exposed these cells to the TLR4 agonist LPS in vitro. As shown in Figure 1G, LPS increased the expression of known target genes in cultured pancreatic pericytes. Thus, our analysis revealed an active TLR/IL-1R pathway in pancreatic pericytes, similar to pericytes in other tissues (34, 37, 56).

### Pericytes express cytokines in a MyD88-dependent manner.

Next, we interfered with the pericytic inflammatory response by selectively inhibiting MyD88, the canonical adaptor of the TLR/IL-1R pathway. Pancreatic pericytes expressed significantly higher levels of *Myd88* than did the other analyzed pancreatic cell populations (Supplemental Figure 1). To delete this adaptor, *Myd88^fl^* mice (57) were crossed with the *Nkx3-2*-Cre line (58), which selectively targets mural cells, primarily pericytes, but not other pancreatic cell types, including epithelial, endothelial, and immune cells (Supplemental Figure 1) (41, 42, 44, 55), to generate Δ*MyD88*^Peri^ (*Nkx3-2*-Cre;*Myd88^fl/fl^*) mice. Notably, all the mice used in this study were fed regular chow, housed in a specific pathogen–free barrier facility, and did not display any indication of infection. As expected, while *Myd88* expression was lost in the pancreatic pericytes of Δ*MyD88*^Peri^ mice, its expression was preserved in pancreatic endocrine and immune cells (Figure 1H and Supplemental Figure 1). To determine the requirement of MyD88 for pericytic cytokine production in vivo, we purified pancreatic pericytes from Δ*MyD88*^Peri^ and control mice based on fluorescence labeling and profiled their transcriptome. RNA-seq analysis revealed that the loss of MyD88 resulted in the upregulation of 6 genes and the downregulation of 55 genes in pancreatic pericytes, including *Myd88* itself and the cytokines *Cxcl1*, *Cxcl13*, *Cxcl12*, and *Il6* (Figure 1I and Supplemental Table 1). Thus, the canonical TLR/IL-1R pathway is required for cytokine production by pancreatic pericytes. Furthermore, our analyses point to the basal activity of the TLR/IL-1R pathway in healthy pancreatic pericytes, which is required for proper cytokine production.

### Pericytic MyD88 activity regulates the number of islet immune cells.

**Figure 2:**
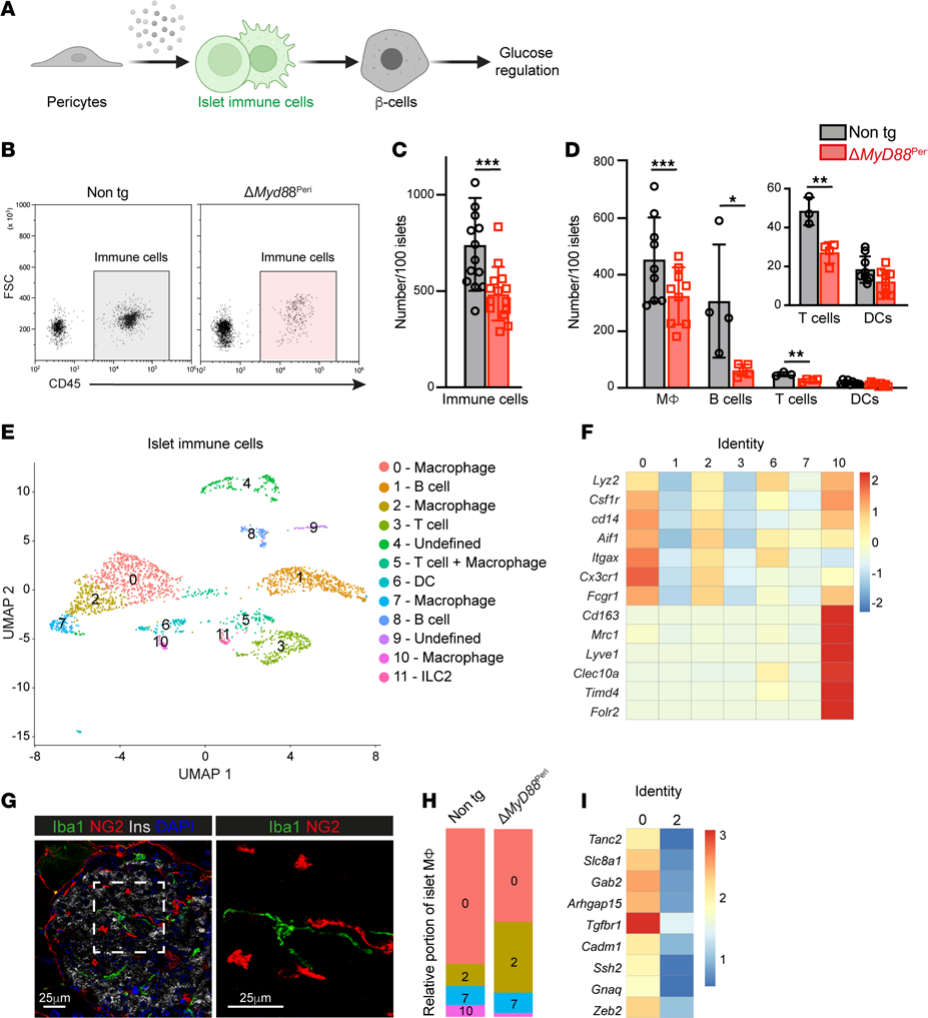
Loss of pericytic MyD88 interferes with the number and phenotype of islet immune cells. Fifteen-week-old Δ*MyD88*^Peri^ (red) and nontransgenic littermates (Cre-negative; ‘‘non tg’’; gray) male mice were analyzed. (**A**) A graphical model of the study’s hypothesis, with immune cells highlighted in green. (**B**) Representative dot plot showing immune cells (CD45^+^ cells) among the dispersed islet cells. (**C** and **D**) Bar diagrams (mean ± SD) showing the total number of immune cells (CD45^+^; **C**), macrophages (MФ; CD45^+^CD11c^+^CD64^+^; **D**), B cells (CD45^+^CD19^+^; **D**), T cells (CD45^+^CD3^+^; **D**), and DCs (CD45^+^CD11c^+^CD64^–^; **D**) in 100 isolated islets. *P < 0.05, **P < 0.001, ***P < 0.005 (unpaired, 2-tailed Student’s *t* test) compared with nontransgenic samples. Each dot represents a single sample. *n* = 4–12. (**E**, **F**, **H**, and **I**) scRNA-seq analysis of islet immune cells from Δ*MyD88*^Peri^ and nontransgenic mice. Shown are UMAP visualization and Seurat clusters with cell annotation (**E**), a heatmap representative of selected macrophage marker expression (**F**), the relative portion of the different macrophage clusters (**H**), and a heatmap representative of selected differential gene expression in cells of clusters 0 and 2 (**I**). (**G**) Immunofluorescence analysis of nontransgenic mouse pancreatic tissue sections for the macrophage marker Iba1 (green), the pericytic marker NG2 (red), insulin (white), and DAPI (blue). The right panel shows a higher magnification of the area framed in the white box on the left panel. Scale bars: 25 μm.

To test our hypothesis further, we aimed to determine the role of pericytes in the regulation of islet inflammation (Figure 2A). To this end, we interfered with the pericytic inflammatory response by selectively inhibiting MyD88 in these cells and analyzed the resulting effects on islet immune cells. Islets isolated from Δ*MyD88*^Peri^ (*Nkx3-2*-Cre;*Myd88^fl/fl^*) mice had fewer immune cells than did those isolated from littermate controls (*Myd88^fl/fl^*; Figure 2, B and C). Next, we quantified the main immune cell populations in healthy islets using flow cytometry (Figure 2D and Supplemental Figure 2). Δ*MyD88*^Peri^ islets had significantly fewer macrophages, B cells, and T cells. Notably, the B cell number was one-fifth of that of the control (Figure 2D). In contrast, the sizes of the corresponding immune cell populations in the blood and spleen were comparable between transgenic and nontransgenic mice (Supplemental Figure 2).

### Abnormal composition and phenotype of immune cells in ΔMyD88^Peri^ islets.

To further define the potential differences between islet immune cells from Δ*MyD88*^Peri^ and those from nontransgenic mice, we performed scRNA-seq analysis of these cells. Macrophages (clusters 0, 2, 7, and 10), DCs (cluster 6), B cells (clusters 1 and 8), T cells (cluster 3), and a small population of type 2 innate lymphoid cells (ILC2s; cluster 11) were detected in the islets (Figure 2E and Supplemental Table 2). The presence of the 4 islet macrophage populations was consistent with previous reports on the heterogeneity of these cells (22, 59, 60). Based on their transcriptome, the macrophages in cluster 10 resembled M2 macrophages (expressing *Cd163*, *Mrc1* [CD206], *Lyve1*, *Clec10a* [CD301], *Timd4*, and *Folr2*), whereas the cells in group 7 did not express any of the known macrophage subtype markers (Figure 2F and Supplemental Table 2). Cells in clusters 0 and 2 represent classical M1-like macrophages (expressing *Lyz2*, *Csf1r*, *Cd14*, *Fcgr1* [CD64], *Itgax* [CD11c], *Aif1* [Iba1], *Cx3cr1*, and *Ccr2*; Figure 2F) (22, 60). M2-like macrophages (cluster 10) expressed *Igf1*, and classical M1-like macrophages (clusters 0 and 2) and DCs (cluster 6) expressed *Il1b* (CD11c^+^ cells; Supplemental Figure 2). Thus, islet macrophage subpopulations also differ in the expression of secreted factors shown to affect β cells (8, 26). Classical macrophages (identified as Iba1^+^ cells), which encompass most islet macrophages (22, 60), are located within the islets in proximity to both β cells and pericytes (Figure 2, G and H).

As changes in macrophage composition are associated with destructive islet inflammation (8), we compared the relative proportions of the 4 macrophage populations in Δ*MyD88*^Peri^ and the control islets. Most of the macrophages in the nontransgenic islets were grouped into cluster 0 when their relative proportions were lower in the transgenic islets (72% vs. 50%; Figure 2H). In contrast, the proportion of macrophages in cluster 2 was higher in Δ*MyD88*^Peri^ islets (38%) than in control (10%; Figure 2H). As detailed above, macrophages belonging to these 2 clusters expressed similar markers of classical macrophages; however, cells in cluster 0 expressed an array of genes not expressed by cells in cluster 2 (Figure 2I and Supplemental Table 2). Among these differentially expressed genes are *Tgfbr1* and *Zeb2*, which are associated with tissue-specific macrophage differentiation (61, 62). Thus, the cells in clusters 0 and 2 may represent macrophages at different differentiation stages when Δ*MyD88*^Peri^ islets are potentially populated with a higher proportion of immature macrophages.

### Pericytic MyD88 is required for β cell function and glucose regulation.

**Figure 3:**
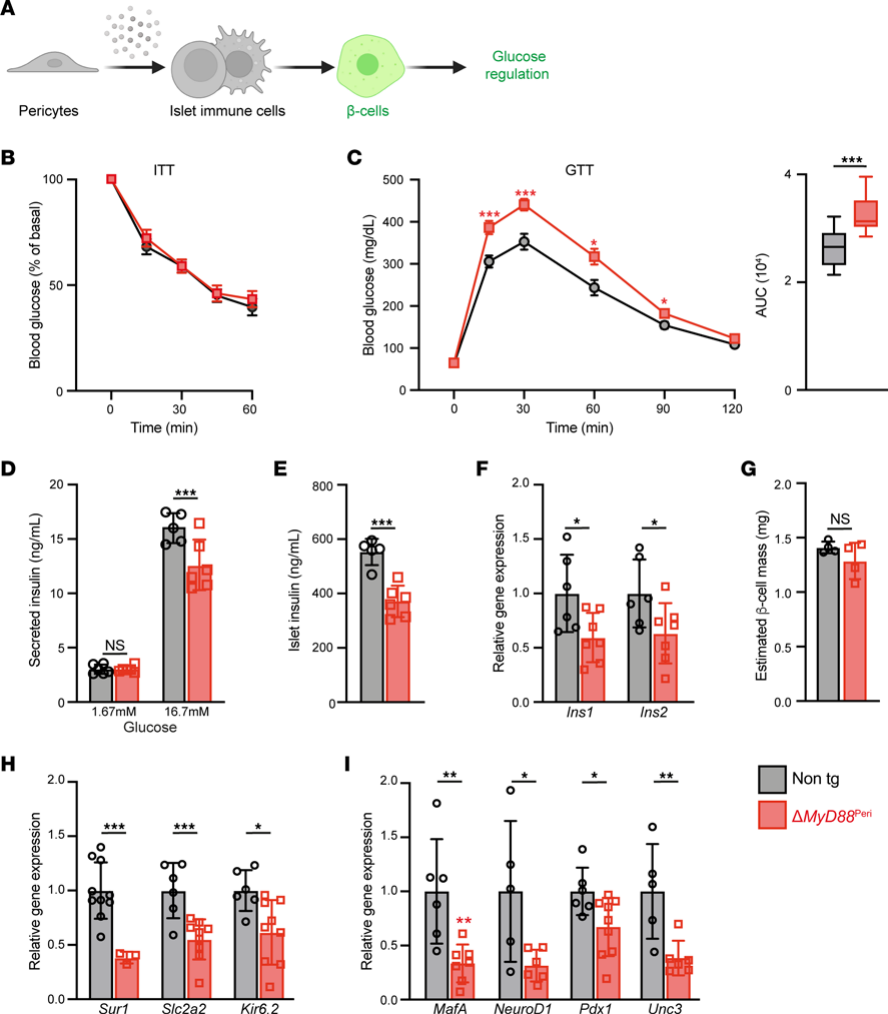
Loss of pericytic MyD88 causes β cell dedifferentiation and glucose intolerance. Fifteen-week-old Δ*MyD88*^Peri^ (red) and nontransgenic littermates (Cre-negative; ‘‘non tg’’; gray) male mice were analyzed. (**A**) Graphical model of the study hypothesis, with β cells highlighted in green. (**B**) Intraperitoneal insulin tolerance test (ITT). The mean (± SEM) blood glucose levels are presented. *n* = 8–10. (**C**) Intraperitoneal glucose tolerance test (IPGTT). Shown are mean (± SEM) blood glucose levels (left) and area under the curve (AUC, right). *n* = 9–12. (**D**) Bar diagram (mean ± SD) showing the glucose-stimulated insulin secretion (GSIS) of isolated islets. *n* = 4–5. (**E**) Bar diagram (mean ± SD) showing the insulin content of the isolated islets. *n* = 4–5. (**F**, **H**, and **I**) Bar diagrams (mean ± SD) showing β cell gene expression analyzed by qPCR. The average levels in the control islets were set to 1. *n* = 5–9. (**G**) Bar diagrams (mean ± SD) showing comparable β cell mass in transgenic and control mice. *n* = 4. *P < 0.05, **P < 0.01, ***P < 0.005; NS, not significant (unpaired, 2-tailed Student’s *t* test) compared to control.

To test our hypothesis further, we examined whether interfering with pericyte-regulated islet inflammation affects β cell function and glucose homeostasis (Figure 3A). Δ*MyD88*^Peri^ and littermate control adult mice displayed comparable weight and basal glucose levels (Supplemental Figure 3). Previous studies have demonstrated that *Nkx3-2*-Cre expression alone does not affect the response to glucose, and is mainly restricted to cells in the gastrointestinal and skeletal systems, with no targeting of hepatic pericytes (41, 58, 63). As expected, Δ*MyD88*^Peri^ and control mice displayed comparable insulin sensitivity (Figure 3B). However, Δ*MyD88*^Peri^ mice (both females and males) exhibited an impaired response to glucose challenge (Figure 3C and Supplemental Figure 3). Thus, our analysis indicated that pancreatic pericytic MyD88 is required for glucose regulation during homeostasis.

Next, we aimed to determine the underlying causes of the impaired glucose response in mice lacking pericytic MyD88. Vascular coverage and pericyte density were comparable between the transgenic and control islets (Supplemental Figure 4). Nevertheless, to study β cell function independently of blood flow, we measured glucose-stimulated insulin secretion (GSIS) in islets isolated from Δ*MyD88*^Peri^ and control mice. While basal insulin secretion was intact, islets lacking pericytic MyD88 secreted less insulin in response to glucose challenge (Figure 3D). This impaired insulin secretion correlated with decreased insulin levels in Δ*MyD88*^Peri^ islets (Figure 3E) and lower expression of the insulin-encoding genes *Ins1* and *Ins2* (Figure 3F). Thus, the lack of pericytic MyD88 affects insulin production and subsequent secretion.

To further define how β cells are affected in Δ*MyD88*^Peri^ mice, we analyzed their phenotype. The β cell mass was unaffected in Δ*MyD88*^Peri^ mice (Figure 3G). In agreement with these findings, transgenic islets did not upregulate the β cell stress genes *Atf4* and *Ddit3* (*Chop*) (Supplemental Figure 4). Furthermore, islet cytoarchitecture and β-to-α cell ratios were similar in transgenic and control mice, as were the *Gcg* and *Sst* expression levels (Supplemental Figure 4). However, Δ*MyD88*^Peri^ islets expressed significantly lower levels of genes encoding components of the GSIS machinery, including *Slc2a2* (Glut2), *Sur1*, and *Kcnj11* (Kir6.2) (Figure 3H). The expression levels of *MafA*, *NeuroD1*, *Pdx1*, and *Ucn3*, which are all required for the mature functional β cell phenotype, were lower in the Δ*MyD88*^Peri^ islets (Figure 3I). Thus, our analysis points to β cell dedifferentiation in the absence of pericytic MyD88 activity.

Next, we investigated whether pericytic MyD88 deficiency influences postnatal β cell development. During the postnatal period, transgenic and control pups exhibited comparable weight gain and pancreatic mass (Supplemental Figure 5). Furthermore, islet morphology and pancreatic insulin content were similar between Δ*MyD88*^Peri^ and littermate control pups (Supplemental Figure 5). Notably, while adult transgenic mice were glucose intolerant (Figure 3C), the glucose responsiveness of pre-adult Δ*MyD88*^Peri^ mice (6 and 10 weeks of age) was comparable to control (Supplemental Figure 5). These findings collectively indicate that the loss of pericytic MyD88 does not impede β cell development and that Δ*MyD88*^Peri^ mice develop glucose intolerance in adulthood.

In conclusion, pericytic MyD88 is required for glucose regulation by supporting the mature β cell phenotype and insulin production during adulthood, potentially by regulating islet inflammation.

### Cxcl1 rescued the β cell phenotype and glucose response of ΔMyD88^Peri^ mice.

**Figure 4:**
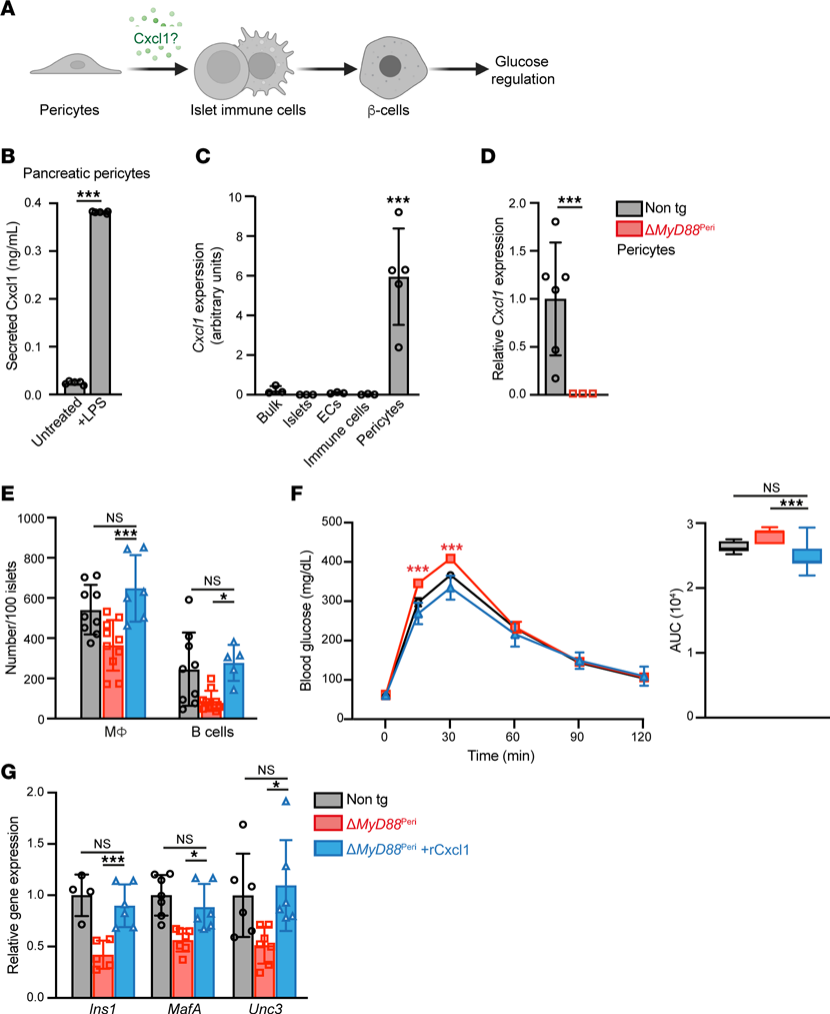
Cxcl1 treatment rescues the glucose intolerance in Δ*MyD88*^Peri^ mice. (**A**) A graphical model of the study’s hypothesis, with Cxcl1 highlighted in green. (**B**) Cultured neonatal pancreatic pericytes were either treated with LPS (right) or left untreated (left), and their supernatant was collected after 48 hours. Bar diagrams (mean ± SD) showing Cxcl1 protein concentration in the supernatant. *n* = 5. ***P < 0.005 (unpaired, 2-tailed Student’s *t* test) compared with the untreated group. Each dot represents a single sample. (**C**) Bar diagram (mean ± SD) showing the results of qPCR analysis of *Cxcl1* transcripts in the indicated pancreatic cell types (as detailed in Figure 1, the average levels in islets were set to 1). *n* = 3–6. ***P < 0.005 (unpaired, 2-tailed Student’s *t* test) relative to the islets. Each dot represents a single sample. (**D**) Bar diagram (mean ± SD) shows qPCR analysis of *Cxcl1* transcripts in pancreatic pericytes of Δ*MyD88*^Peri^ mice (red) and nontransgenic (“non tg”; gray; the average was set to 1) mice. *n* = 3–6. ***P < 0.005 (unpaired, 2-tailed Student’s *t* test) compared with nontransgenic mice. Each dot represents a single sample. (**E**–**G**) Analyses of Δ*MyD88*^Peri^ mice treated with recombinant Cxcl1 (rCxcl1; blue), PBS-treated Δ*MyD88*^Peri^ (red), or PBS-treated nontransgenic (black line and gray bars) 15-week-old mice, 1 week after treatment. (**E**) Bar diagram (mean ± SD) showing the number of macrophages (MФ; CD45^+^CD64^+^ cells) and B cells (CD45^+^CD19^+^ cells) in 100 islets. *n* = 6–10. (**F**) IPGTT. Shown are the mean (± SEM) blood glucose levels (left) and the AUC (right). *n* = 7–8. (**G**) Bar diagrams (mean ± SD) showing expression of indicated genes in isolated islets. *n* = 4–7. *P < 0.05, ***P < 0.005; NS, not significant (1-way ANOVA with Tukey’s post hoc test). Each dot represents a single sample.

Our analysis indicated that pericytes regulate islet inflammation and glucose homeostasis. Therefore, we aimed to elucidate the potential underlying molecular mechanism by focusing on a single pericytic cytokine (Figure 4A). A potential candidate is CXCL1, which is expressed in both human and mouse pericytes (Figure 1, B and C). Pancreatic pericytes secrete Cxcl1 (Figure 4B) and are the primary source of this cytokine in the pancreas (Figure 4C). Importantly, our analysis indicated that pericytic Cxcl1 levels depended on the TLR4/MyD88 pathway (Figure 1, G and I, and Figure 4, B and D). Thus, we hypothesized that the loss of pericytic Cxcl1 contributes to MyD88-dependent abrogation of islet inflammation and β cell function.

To test this hypothesis, we analyzed the impact of exogenous Cxcl1 on islet immune cells of Δ*MyD88*^Peri^ mice. As shown in Figure 4E, recombinant Cxcl1 (rCxcl1) administration increased the number of macrophages and B cells within transgenic islets to the level observed in nontransgenic control islets. This finding suggests that pericytic Cxcl1 regulates the number of immune cells in the islets, and the decrease in Cxcl1 in Δ*MyD88*^Peri^ mice likely contributes to the abnormal islet inflammation observed in these mice.

Next, we tested the contribution of Cxcl1 loss to glucose intolerance in Δ*MyD88*^Peri^ mice. Treatment with rCxcl1 rescued the glucose response of transgenic animals, which became comparable to that of nontransgenic control mice (Figure 4F). Similar treatments did not significantly improve the glucose response in wild-type mice (Supplemental Figure 6) (64). Moreover, administration of rCxcl1 did not influence food intake or weight gain in Δ*MyD88*^Peri^ mice for up to 4 weeks after treatment. To determine whether the observed reversal of glucose intolerance is associated with the correction of the β cell phenotype in transgenic mice, we analyzed the islets of rCxcl1-treated Δ*MyD88*^Peri^ mice for genes associated with β cell maturity. As shown in Figure 4G, rCxcl1 treatment increased the expression of *Ins1*, *MafA*, and *Unc3* in Δ*MyD88*^Peri^ islets, making their levels comparable to those in nontransgenic controls. Thus, the low levels of pericytic Cxcl1 in Δ*MyD88*^Peri^ mice likely contributed to their β cell failure and glucose intolerance.

In conclusion, our analysis suggested that pericytes produce Cxcl1 in a MyD88-dependent manner to regulate islet inflammation and support the β cell phenotype and glucose regulation.

### IL-1β production by islet macrophages depends on pericytic MyD88 and Cxcl1.

**Figure 5:**
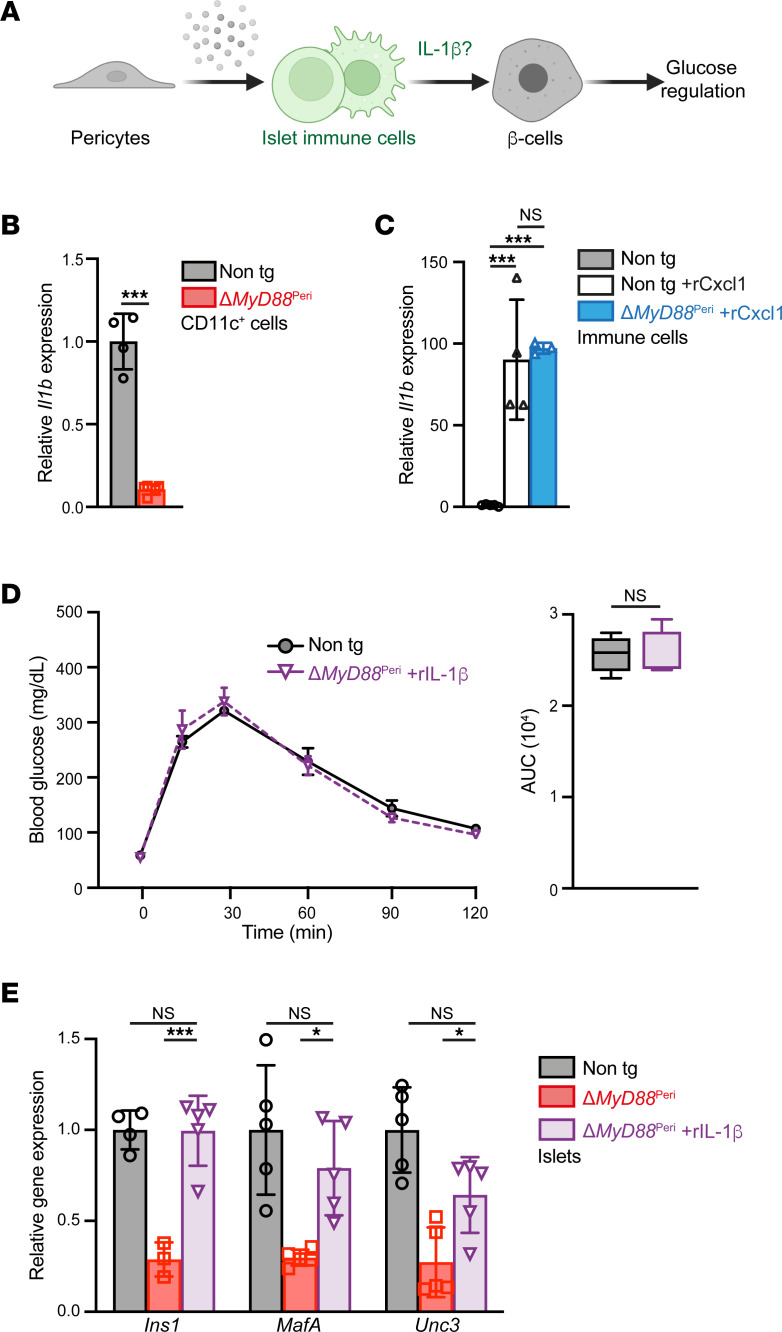
Pericytic MyD88 is required for immune IL-1β production. (**A**) A graphical model of the study’s hypothesis, with IL-1β highlighted in green. (**B**) Bar diagram (mean ± SD) showing reduced *Il1b* expression in pancreatic macrophages and DCs of transgenic mice. Shown is a qPCR analysis of *Il1b* transcripts in CD11c^+^ pancreatic cells from Δ*MyD88*^Peri^ (red) and nontransgenic (gray) 15-week-old mice. *n* = 4–5. ***P < 0.005 (unpaired, 2-tailed Student’s *t* test). Each dot represents a single sample. (**C**) Cxcl1 induces *Il1b* expression in pancreatic cells in vivo. Δ*MyD88*^Peri^ (blue) and nontransgenic (empty bars) adult mice were treated with mouse rCxcl1 (1 μg/g body weight), and their pancreatic immune cells (CD45^+^ cells) were analyzed 1 week later and compared to those of PBS-treated nontransgenic mice (gray bars, the average was set to 1). Bar diagrams (mean ± SD) showing *Il1b* expression analyzed by qPCR. *n* = 4–6. ***P < 0.005; NS, not significant (1-way ANOVA with Tukey’s post hoc test). Each dot represents a single sample. (**D**) IPGTT of rIL-1β–treated Δ*MyD88*^Peri^ (purple) and PBS-treated nontransgenic (black line and gray bars) 15-week-old mice 1 day after treatment. The mean (± SEM) blood glucose levels (left) and area under the curve (AUC, right) are shown. *n* = 4–5. NS, not significant (1-way ANOVA with Tukey’s post hoc test). (**E**) qPCR analysis of islets isolated from rIL-1β–treated Δ*MyD88*^Peri^ (purple), PBS-treated Δ*MyD88*^Peri^ (red), and nontransgenic (gray) 15-week-old mice 1 day after treatment. Bar diagrams (mean ± SD) show expression of indicated genes. *n* = 4–6. *P < 0.05, ***P < 0.005; NS, not significant (1-way ANOVA with Tukey’s post hoc test). Each dot represents a single sample.

Next, we aimed to determine how pericytic MyD88 deficiency affects the ability of macrophages to support β cells (Figure 5A). As macrophages were shown to directly affect β cell function through the secretion of IL-1β (8), we tested the hypothesis that the loss of pericytic MyD88 interfered with the production of this cytokine. To determine whether *Il1b* expression was affected in Δ*MyD88*^Peri^ mice, we compared its expression in nontransgenic and transgenic pancreatic macrophages and DCs (i.e., CD11c^+^ cells). As shown in Figure 5B, *Il1b* expression in pancreatic CD11c^+^ cells from Δ*MyD88*^Peri^ mice was an order of magnitude lower than that in cells from control mice, supporting our hypothesis.

Cxcl1 induces IL-1β production by bone marrow–derived macrophages (65). To test whether Cxcl1, expressed by pancreatic pericytes in a TLR/MyD88-dependent manner, has similar effects on pancreatic immune cells in vivo, we treated mice with this cytokine. As shown in Figure 5C, rCxcl1 treatment boosted *Il1b* expression in pancreatic immune cells. Interestingly, rCxcl1 had a similar effect on cells isolated from wild-type and transgenic mice (Figure 5C). Thus, we suggest that pericytes regulate the local production of IL-1β by islet macrophages and DCs in healthy individuals in a MyD88-dependent manner, potentially through the production of Cxcl1.

### IL-1β rescued the glucose intolerance of ΔMyD88^Peri^ mice.

Next, we tested the potential contribution of IL-1β to glucose intolerance in the Δ*MyD88*^Peri^ mice. To this end, we injected transgenic animals with recombinant murine IL-1β (rIL-1β). One day after treatment, the glucose response of treated transgenic animals was comparable to that of nontransgenic controls (Figure 5D). Similar treatment with rIL-1β did not affect the glucose response in wild-type mice (Supplemental Figure 7). To define potential effects on β cell gene expression, we analyzed rIL-1β–treated Δ*MyD88*^Peri^ islets. Similarly to Cxcl1, rIL-1β treatment in vivo increased the expression of *Ins1*, *MafA*, and *Unc3* in Δ*MyD88*^Peri^ islets, making their levels comparable to those in nontransgenic controls (Figure 5E). An increase in the expression of these 3 genes was also observed when islets isolated from Δ*MyD88*^Peri^ mice were treated with rIL-1β in culture, suggesting a direct effect on β cells (Supplemental Figure 7). Thus, lower levels of IL-1β in Δ*MyD88*^Peri^ mice likely contributed to β cell failure and glucose intolerance. In conclusion, our analysis suggested that pericytes produce Cxcl1 in a MyD88-dependent manner to support the β cell phenotype and glucose regulation, likely by facilitating IL-1β production by islet macrophages and DCs.

## Discussion

Here, we showed that pericytes play a crucial role in regulating islet inflammation to maintain glucose homeostasis. Interference with the pericytic TLR/MyD88 pathway impaired cytokine production in healthy mice, leading to aberrant islet inflammation. The loss of pericytic MyD88 affected the number and phenotype of islet immune cells and impaired IL-1β production by islet macrophages and DCs. Aberrant pericyte-orchestrated islet inflammation was associated with impaired insulin production and secretion, loss of the β cell mature phenotype, and glucose intolerance. Treating mice with the MyD88-dependent cytokine Cxcl1 increased islet immune cell number and IL-1β production, restoring their β cell phenotype and glucose response. Similarly, treating pericytic MyD88-deficient mice with IL-1β improved their insulin expression and glucose response. Thus, we suggest that pericytes locally secrete cytokines to modulate islet immune cells and maintain β cell function and glucose homeostasis.

The loss of pericytic MyD88 and dependent cytokines affected multiple dimensions of islet inflammation, including the number and composition of immune cells in the islets and the phenotype of these cells. Pericytes have been shown to modulate the inflammatory response to tissue damage, allowing proper regeneration (34, 35, 66). Our analysis suggested that pericytes play a similar role in maintaining islet homeostasis without apparent tissue damage or infection. This activity somewhat resembles the role of pericytes in maintaining blood-retina barrier integrity (35, 66). However, how pericyte-orchestrated islet inflammation is affected by stress (as obesity or aging) remains to be elucidated.

Our findings suggest that the loss of pericytic MyD88 has a localized impact on the size of islet immune populations, particularly macrophages and B cells. Reducing the number of immune cells may have direct and indirect effects on islet function. For example, macrophages support β cells by producing various secreted factors (7, 12, 14, 17, 26). Therefore, the decreased number of macrophages in Δ*MyD88*^Peri^ islets is likely to result in lower levels of these supportive factors, directly impacting β cells. In contrast, although islets of healthy individuals are populated by lymphocytes, the precise role of these cells in glucose regulation remains unclear (67, 68). Consequently, the potential impact of T and B cell deficiency upon loss of pericytic MyD88 is unclear.

Although our analysis focused on Cxcl1, other pericytic cytokines may also play a role in glucose regulation. For example, Cxcl12 supports β cell survival in a mouse model of type 1 diabetes and recruits macrophages to promote β cell regeneration after damage (12, 49), while IL-6 has been shown to directly enhance insulin secretion (51, 53, 54). Moreover, pericytes may promote immune cell infiltration by regulating vascular permeability and dilation (32). Thus, pericytes locally regulate islet inflammation in a MyD88-dependent manner, including through cytokine secretion, to support β cell function in health and glucose regulation.

Similar to other cytokines, IL-1β exhibits context- and locally dependent activities (69). It has both supportive and destructive effects on β cell function, causing dysfunction with prolonged exposure and inducing insulin secretion with acute exposure (27, 28, 70). Brain and peritoneal macrophages secrete IL-1β to promote feeding-dependent insulin secretion (71, 72). In mice unable to respond to this cytokine, glucose intolerance arises due to β cell dysfunction (70, 73, 74). Our findings suggest that pericytes regulate the local production of IL-1β by islet macrophages and DCs in healthy individuals, thus facilitating their support of β cell function. The impairment of this regulation in a diseased state requires further exploration.

Changes in islet inflammation correspond to T2D, potentially reflecting adaptation to increased metabolic demand rather than malfunction (8), although prolonged islet inflammation can be harmful. Targeting islet inflammation appears promising for diabetes treatment (8), and our study highlights its local regulation. However, whether changes in pericyte inflammatory activity underlie the transformation of islet inflammation into a harmful process that drives diabetes remains unclear. Nevertheless, our study introduced pericyte-orchestrated islet inflammation as a target for new therapeutic interventions aimed at improving β cell function and glucose regulation.

## Methods

### Sex as a biological variable.

Our study examined male and female animals, and similar findings are reported for both sexes.

### Mice.

Mice were maintained on a C57BL/6 background. *Nkx3-2*-Cre (Nkx3-2^tm1(cre)Wez^) mice (58) were a gift from Warren Zimmer (Texas A&M University, College Station, Texas, USA). *R26*-YFP [B6.129X1-*Gt(ROSA)26Sor^tm1(EYFP)Cos^*/J] and *Myd88^fl^* [B6.129P2(SJL)-*Myd88^tm1Defr^*/J] (57) mice were obtained from the Jackson Laboratory. Wild-type mice were purchased from Envigo Ltd. Mice were housed under specific pathogen–free conditions in a 22°C temperature-controlled room and a regular 12-hour light/dark cycle in individually ventilated cages. The mice were fed standard chow ad libitum and had free access to water. All the experiments were performed on sex- and age-matched littermate mice. Mice were analyzed at the indicated ages.

### Mouse treatments.

Glucose tolerance tests (GTTs) were conducted by intraperitoneal injection of 2 mg/g dextrose (Sigma-Aldrich, D9434) into mice following overnight fasting. Insulin tolerance tests (ITTs) were performed by intraperitoneal injection of 0.75 U/kg insulin (Eli Lilly, HI 0210) after a fasting period of 6 hours. Blood glucose levels in the tail vein were measured using Contour glucometers (Bayer). For Cxcl1 treatment, mice were intraperitoneally injected with either carrier-free murine rCxcl1 (1 μg/g body weight; R&D Systems, 453KC) diluted in PBS or PBS alone and analyzed. For IL-1β treatment, mice received an intraperitoneal injection of carrier-free murine rIL-1β (100 ng/kg body weight; R&D Systems, 401ML) diluted in PBS or PBS alone and analyzed 1 day after the injection. For food consumption analysis, mice were individually housed in separate cages for 24 hours, and the remaining pellets were weighed to determine the quantity consumed by each mouse.

### Islet isolation.

Cold collagenase P solution (0.8 mg/mL; Roche, 11213865001) was injected into the pancreas of euthanized mice using a 30-G needle. The pancreas was then removed and incubated with collagenase P at 37°C for 10–11 minutes, followed by termination with cold RPMI medium and subsequent centrifugation. The resulting pancreatic pellet was washed, strained, and subjected to density gradient separation using Histopaque-1119 (Sigma-Aldrich, 11191) and RPMI media, followed by another round of centrifugation. The islets were then collected, washed, and handpicked for subsequent analyses.

### Cell isolation.

To isolate pancreatic cells, pancreatic tissues were dissected and digested with collagenase P (0.4 mg/mL) and DNase I (0.1 ng/mL; Sigma-Aldrich, D5025) diluted in HBSS, followed by incubation at 37°C with agitation. The tissue digestion was terminated using ice-cold HBSS, followed by centrifugation and filtration through a 70-μm strainer to yield a single-cell suspension. Cells were resuspended in PBS containing FBS and EDTA and filtered through a 35-μm strainer. For specific cell type isolation, pancreatic pericytes were sorted by flow cytometry, while pancreatic endothelial and immune cells were sorted either through flow cytometry or magnetic separation, as detailed below. Islet immune cells were isolated by dispersing double-hand-picked islets in Accutase (Sigma-Aldrich, A6964) for 5 minutes. Blood immune cells were obtained from tail vein blood collected in EDTA tubes and then separated using a Lymphoprep (StemCell Technologies, 07801) density gradient. Splenic cells were isolated by injecting the tissue with collagenase D (1 mg/mL; Roche, 11088866001), followed by slicing, incubating, and mincing. The pellet resulting from the splenic process was resuspended in ACK buffer (8 g NH_4_Cl and 1 g KHCO_3_ dissolved in 1 liter of double-distilled water, pH 7.2–7.4) for erythrocyte lysis.

### Flow cytometry.

For sorting and analysis, immune and endothelial cells were identified by surface markers. To this end, dispersed cells were incubated with an Fc blocker, followed by antibody staining (Supplemental Table 3). Next, the cells were incubated with DAPI (200 ng/mL; Sigma-Aldrich, D9542), which was used to identify and exclude dead cells. Pericytes were identified based on their fluorescence labeling in *Nkx3-2*-Cre;*R26*-YFP mice. Cells were analyzed using a Cytoflex (Beckman Coulter) and the data were processed using Kaluza software (Beckman Coulter) or collected using FACSAria III cell sorters (BD).

### Magnetic separation.

Cell purification was achieved using magnetic-activated cell sorting (MACS) following the manufacturer’s guidelines. Briefly, cells suspended in cold PBS supplemented with 0.5% BSA and 2 mM EDTA were incubated with specific microbeads (Miltenyi Biotec; CD31, 130097418; CD45, 13005230; CD11c, 130125835), washed, and centrifuged. The cells were then applied to a MACS column (Miltenyi Biotec, 130-042-401) in a MACS separator (Miltenyi Biotec, 130-042-301). After removal from the separator, the columns were washed, and the magnetically labeled cells were collected as the positive fraction.

### Insulin measurements.

For the GSIS assay, isolated islets were initially incubated for 30 minutes in a modified glucose-free RPMI medium supplemented with 25 mM HEPES, 0.1% BSA, and 1.67 mM glucose at pH 7.4. After preincubation, the medium was replaced with fresh medium. In each assay, 10 uniformly sized islets were carefully selected using a dissecting microscope and transferred in a volume of 5 μL to a well of a 96-well U-bottom plate containing 250 μL of medium with either a low (1.67 mM) or high (16.7 mM) glucose concentration. The islets were incubated for 60 minutes at 37°C and 5% CO_2_, and the media were collected and stored at –80°C. For measuring islet insulin content, islets were transferred into tubes containing 300 μL of 1.5% HCl in 70% ethanol solution, followed by 2 minutes of cell lysis using a TissueLyser II (Qiagen) with two 3-mm metal beads (frequency 1/25 s) and incubated overnight at 4°C. The homogenized samples were neutralized with 1:1 (v/v) of 0.4 M Tris hydrochloride, pH 8. Islet insulin levels were determined using a mouse ultrasensitive insulin ELISA (Alpco, 80-INSMSU-E10) according to the manufacturer’s protocol. For pancreatic insulin content, tissues were resuspended 2 mL of 1.5% HCl in 70% ethanol solution, followed by 2 minutes of cell lysis using a TissueLyser II (Qiagen) with two 3-mm metal beads (frequency 1/25 s). Samples were incubated overnight in 5 mL of 1.5% HCl in 70% ethanol at 4°C. The homogenized samples were then neutralized with 1:1 (v/v) of 1 M Tris hydrochloride, pH 7.5. Protein content was measured using a Pierce BCA Protein Assay Kit (Thermo Fisher Scientific, 23227). Insulin content was measured by Mouse High Range Insulin ELISA (Alpco, 80-INSMSH-E01).

### Gene expression analysis.

For qPCR analysis, RNA was extracted from cells and tissues using PureLink RNA Mini and Micro Kits (Invitrogen, 12183-016 and 12183-018) according to the manufacturer’s protocol and quantified using a NanoDrop Lite Spectrophotometer (Thermo Fisher Scientific). The extracted RNA was transcribed into cDNA using a high-capacity cDNA reverse transcription kit (Applied Biosystems, 4374966). Gene expression levels were detected with TaqMan or SYBR Green assays using the indicated primers and were normalized to the housekeeping gene glyceraldehyde 3-phosphate dehydrogenase (GAPDH) or cyclophilin, respectively (Supplemental Table 4). All assays were conducted in technical duplicate. Transcript levels were measured using a StepOne cycler (Applied Biosystems) and analyzed using the comparative 2^–ΔΔCt^ method.

RNA-seq and scRNA-seq libraries were prepared using the SMART-Seq stranded Kit (Takara Bio, 634442) following the low-input protocol. During cDNA amplification and library amplification, 5 and 14 PCR cycles, respectively, were performed. Libraries were sequenced on a NextSeq 500 platform (Illumina) with a NextSeq 500/550 High Output Kit v2.5 (75 cycles) kit (Illumina). Both library preparation and sequencing were performed at the Genomics Research Unit of the Life Sciences Inter-Departmental Research Facility Unit, Tel Aviv University, Israel.

The RNA-seq data were preprocessed using the Seq2Scince pipeline (75) with the default settings. STAR was used as the aligner (76), HTSeq was used for gene counting (77), and TPM normalization was performed using Salmon (78). DESeq2 (79) was used for the differential gene expression analysis when differentially expressed genes were defined as genes with adjusted *P* values lower than 0.05 and log_2_(fold changes) higher or lower than 0.5 and –0.5, respectively. The results of the differentially expressed gene analysis were plotted using the ggplot2 package in R.

scRNA-seq data were analyzed by the Mantoux Genomics Institute of the Nancy and Stephen Grand Israel National Center for Personalized Medicine, Weizmann Institute of Science. In detail, bioinformatics processing was performed using Cell Ranger v6 (10× Genomics) with default parameters for alignment, filtering, barcode counting, and unique molecular identifier (UMI) counting. The Seurat package in R (80) was used for downstream analysis and visualization. Normalization of the reads was performed using log normalization. Dimension reduction was conducted using principal component analysis (PCA). Clustering was achieved using a K-nearest neighbors (KNN) graph, with visualization and nonlinear dimension reduction accomplished through uniform manifold approximation and projection (UMAP). Marker genes were identified by performing differential expression analysis using the nonparametric Wilcoxon’s rank sum test in accordance with the default methodology in Seurat.

### Immunofluorescence.

For cryosections, pancreatic tissues were fixed in 4% paraformaldehyde for 4 hours, cryoprotected in 30% sucrose, and embedded in OCT compound. Sections (11 μm thick) were then cut using a cryostat (Leica CM1950). For paraffin sectioning, tissues were fixed in paraformaldehyde, washed in PBS, and dehydrated through a graded ethanol series, followed by xylene immersion and paraffin infiltration at 56°C in a Tissue Processor (Leica TP1020). Sections 5 μm in thickness were subsequently cut using a Thermo Fisher Scientific Microtome (HM355S) and mounted on HistoBond+ slides. For antigen retrieval, sections from paraffin-embedded tissues and cryosections were heated in Citra buffer (BioGenex, HK086-9K) in a microwave water bath for 15 minutes. Immunostaining was performed overnight with primary antibodies at 4°C (Supplemental Table 3), followed by 1-hour incubation with fluorescent secondary antibodies at room temperature (Supplemental Table 5). Sections were mounted with Vectashield mounting medium containing DAPI (Vector Laboratories, H-1500). Imaging was conducted using a BZ-9000 BioRevo (Keyence), BZ-X810 BioRevo (Keyence), and an SP8 confocal microscope (Leica).

### Morphometric quantification.

To analyze functional vasculature, mice were intravascularly injected with 100 μL of fluorescently labeled tomato lectin (1 mg/mL; Vector Laboratories, FL-1171). After injection, the pancreas was fixed, sectioned, and immunostained for insulin. Stained sections were imaged using a BZ-9000 BioRevo microscope. The ratio of lectin^+^ to insulin^+^ area was calculated for 100 islets. Islet pericyte coverage was analyzed by immunostaining cryosections for NG2 and insulin (Supplemental Table 3), with the NG2^+^ area proportion calculated per 100 islets (defined as the insulin^+^ area).

To assess the β cell mass, paraffin-embedded pancreatic tissue sections were immunostained for insulin. Sections at least 100 μm apart were counterstained with HCS CellMask Deep Red Stain (Thermo Fisher Scientific, H32721) to label the entire tissue sample. Stained sections were scanned as a whole using an Aperio VERSA (Leica). For each mouse, the insulin^+^ area was quantified and normalized to the total pancreatic area. This ratio was subsequently multiplied by the weight of the pancreas to calculate the β cell mass. To assess the β cell/α cell ratio, paraffin-embedded pancreatic tissue sections were immunostained for insulin and glucagon, and the insulin^+^-to-glucagon^+^ area ratio was calculated for each islet. Morphometric quantifications were performed using ImageJ (NIH).

### Cell culture.

For pancreatic pericyte culture, cells were FACS isolated from mice on postnatal day 5 and cultured in DMEM supplemented with 10% FCS, 1% L-glutamine, and 1% penicillin-streptomycin, as previously described (81). For LPS treatments, pericytes were cultured in a medium either supplemented with 1 μg/mL LPS (Sigma-Aldrich, L4005) or unsupplemented for indicated periods. A mouse CXCL1/KC Quantikine ELISA Kit (R&D Systems, MKC00B) was used to measure Cxcl1 levels in the supernatant. To evaluate the effects of IL-1β on islets in vitro, islets isolated from 4 mice were pooled together and cultured. Each 96-well-plate well contained 50 islets cultured in CMRL (Gibco, 21530027; supplemented with 10% FBS, 1% penicillin-streptomycin, and 1% L-glutamine) either with 50 pg/mL of rIL-1β (R&D Systems, 401ML) or without treatment for 24 hours.

### Statistics.

For all data sets, excluding RNA-seq and scRNA-seq, statistical analyses were performed using an unpaired, 2-tailed Student’s *t* test or 1-way ANOVA with Tukey’s post hoc test, as indicated. Prism software v9 and v10 (GraphPad Software) were used for all the analyses. Differential expression for RNA-seq and scRNA-seq data was analyzed as described above. The Grubbs method was applied to identify and exclude statistically significant outliers (α = 0.05). The results are expressed as the mean ± standard error of the mean (SEM) or mean ± standard deviation (SD). A *P* value of less than 0.05 was considered significant. Box-and-whisker plots were generated using GraphPad Prism v10. In these plots, the box represents the interquartile range (IQR), extending from the 25th percentile (Q1) to the 75th percentile (Q3) when outliers were defined as data points beyond 1.5 times the IQR. The whiskers stretch to the minimum and maximum values of the data set, and the line within the box indicates the median value.

### Study approval.

All the experimental protocols used were approved by the Tel Aviv University Institutional Animal Care and Use Committee (IACUC). The study was conducted in compliance with the ARRIVE guidelines.

### Data availability.

All data generated during this study are included in this published article and its supplementary information files, including the Supporting Data Values file. Raw sequence files are available on ArrayExpress (https://www.ebi.ac.uk/biostudies/arrayexpress) with accession numbers E-MTAB-13639 and E-MTAB-13640.

## Author contributions

AS conducted experiments, analyzed data, and wrote the manuscript. DAN and AE conducted experiments. SO analyzed data. ME, SP, TW, and SMM conducted experiments and analyzed data. RAP supervised the study. LL conceptualized the study, wrote the manuscript, supervised the study, and acquired funding.

## Supplementary Material

Supplemental data

Supplemental table 1

Supplemental table 2

Supporting data values

## Version 1 06/17/2024

In-Press Preview

## Version 2 07/15/2024

Electronic publication
