# Supplementary material for: Beneficial islet inflammation in health depends on pericytic TLR/MyD88 signaling
Source: J Clin Invest. 2024 Jun 17;134(14):e179335. doi: 10.1172/JCI179335 (PMC11245159; doi:10.1172/JCI179335)
Supplement: Supplemental data [file jci-134-179335-s011.pdf]

Supplemental Data

Beneficial islet inflammation in health depends on pericytic TLR/MyD88 signaling

Anat Schonblum, Dunia Ali Naser, Shai Ovadia, Mohammed Egbaria, Shani Puyesky, Alona Epshtein, Tomer Wald, Sophia Mercado-Medrez, Ruth Ashery-Padan and Limor Landsman

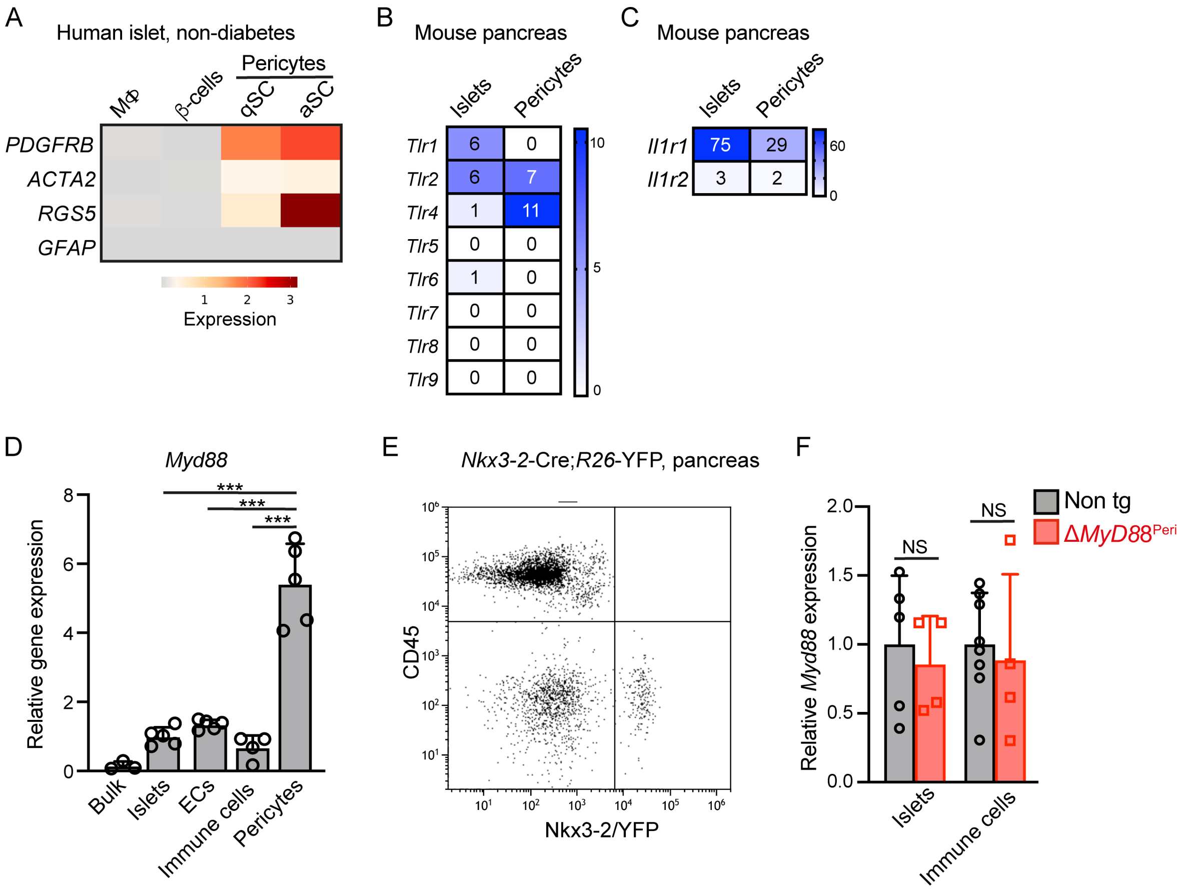

Supplemental Figure 1: Expression of the TLR/IL1R pathway components in the pancreas

A) Heatmap showing relative expression of pericytes and stellate cell markers in macrophages (cluster 10), beta-cells (cluster 3), and pericytes [clusters 5 and 9, originally annotated as 'quiescent stellate (qSC)' and 'activated stellate (aSC)'], employing published scRNA-Seq analysis of islets from healthy human donors (1).

B, C) Heatmap showing relative expression of TLR/IL1R pathway receptors in isolated pericytes and islets, employing a previously published RNAseq analysis of mouse pancreata (2).

D) Bar diagram (mean ± SD) shows qPCR analysis of *Myd88* (E) transcripts in bulk pancreatic tissues, isolated islets (average set to '1'), pancreatic endothelial cells (ECs), pancreatic immune cells and pancreatic pericytes of adult mice. N = 3-5. ***p < 0.005 (Student's t test). Each dot represents a single sample.

E) Representative dot plot showing immune cells ($CD45^+$ cells) in pancreata of YFP^{Peri} (*Nkx3.2*-Cre;*R26*-YFP) mice.

F) Bar diagram (mean ± SD) showing the results of qPCR analysis of *Myd88* transcripts in pancreatic cells. Islets and immune cells ($CD45^+$ cells) were isolated from *Nkx3.2*-Cre;$Myd88^{flox/flox}$ ($\Delta MyD88^{Peri}$; red) and non-transgenic ("non tg"; gray; the average was set to '1') mice. N = 4–8. Each dot represents a single sample.

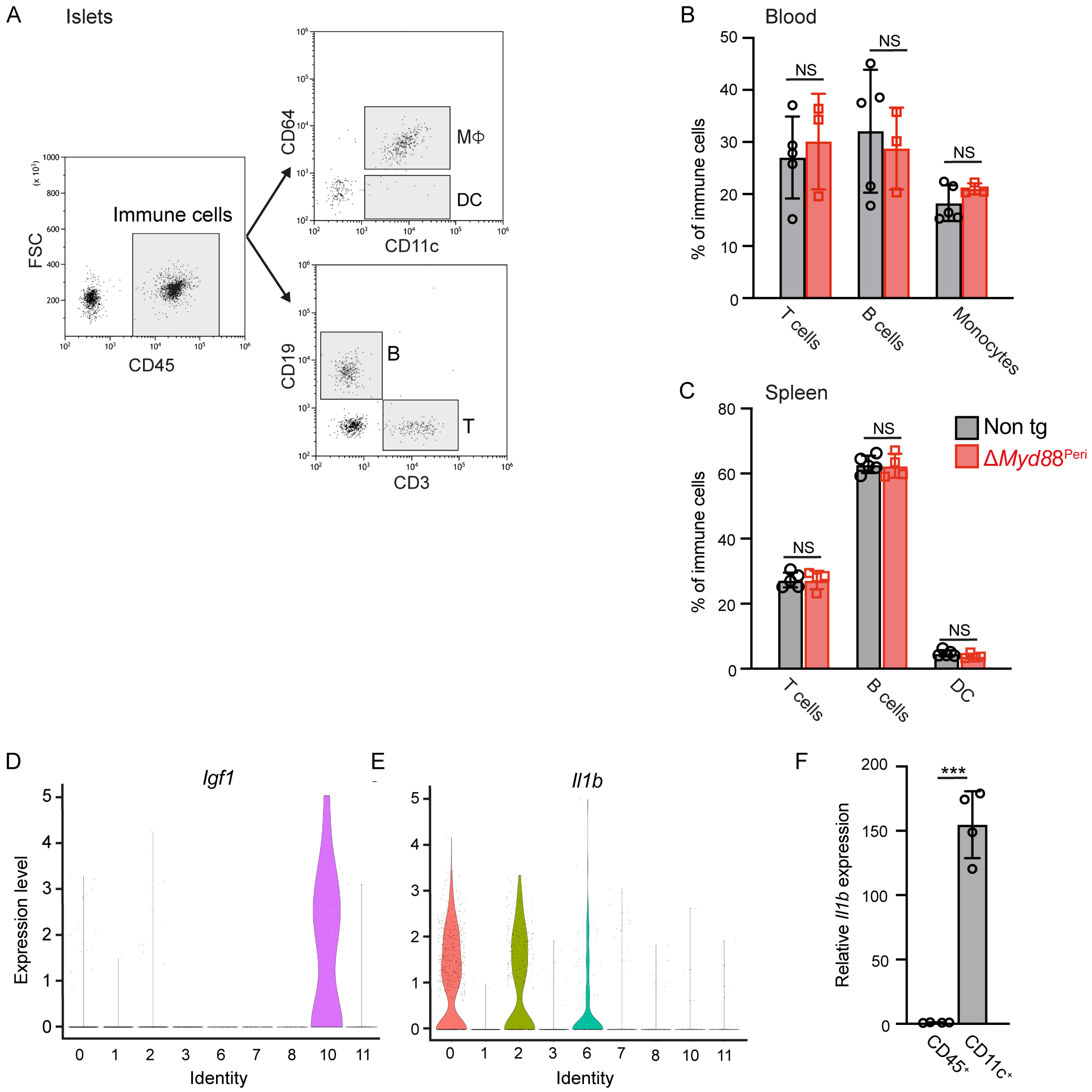

Supplemental Figure 2: Analysis of immune cells in the islet, spleen, and blood of MyD88-deficient mice

15-week-old Δ*MyD88*^{Peri} (red) and non-transgenic (gray) mice were analyzed.
A) Representative dot plots indicating gate used to identify islet cells: Immune cells (Left panel, $CD45^+$ cells), macrophages (MΦ; Top right panel, $CD45^+CD11c^+CD64^+$ cells), DCs (Top right panel, $CD45^+CD11c^+CD64^-$ cells), T cells (Bottom right panel, $CD45^+CD3^+$ cells) and B cells (Bottom right panel, $CD45^+CD19^+$ cells).
B) Bar diagrams (mean ± SD) showing the portion of T cells ($CD3^+$), B cells ($CD19^+$), and monocytes ($CD115^+$) out of total blood immune ($CD45^+$) cells. N = 3-4.
C) Bar diagrams (mean ± SD) showing the portion of T cells ($CD3^+$), B cells ($CD19^+$), and DCs ($CD11c^+$) out of total splenic immune ($CD45^+$) cells. N = 4-5.
D, E) scRNA-seq analysis of islet immune cells. Violin plots showing *Il1b* (E) and *Igf1* (D) expression in the different cell clusters.
F) Bar diagram (mean ± SD) showing the results of qPCR analysis of the *Ilb* transcript in total islet immune cells ($CD45^+$; the average was set to '1') and of the $CD11c^+$ macrophages and DCs from non-transgenic adult mice. N = 4–5. ***$p < 0.005$ (Student's t test). Each dot represents a single sample.

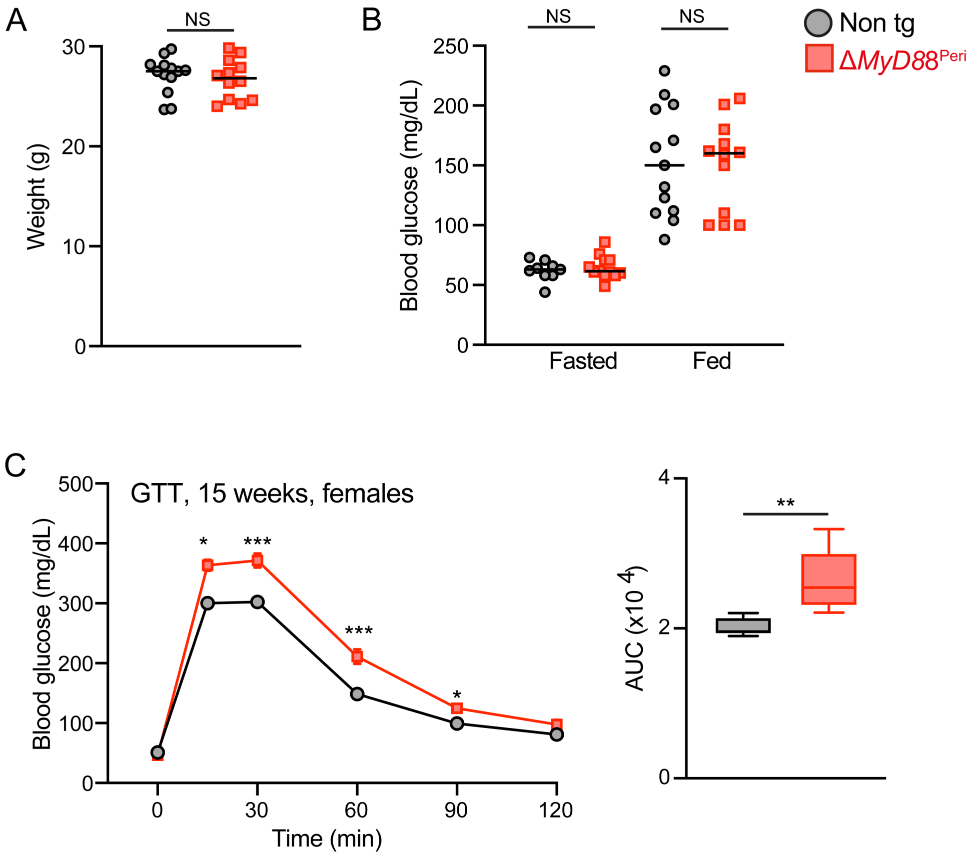

Supplemental Figure 3: Pericytic MyD88-deficient female mice are glucose intolerant

15-week-old Δ*MyD88*^{Peri} (red) and non-transgenic littermates (Cre-negative; ‘‘non tg’’; gray) mice were analyzed.

A) Mean/dot plot shows the body weight of male mice. NS, not significant (Student's t test).

B) Mean/dot plot shows the blood glucose levels of male mice after an overnight fast or upon ad-lib feeding. NS, not significant (Student's t test).

C) Intraperitoneal glucose tolerance test (IPGTT) analysis of female mice. Shown are mean (± SEM) blood glucose levels (left) and area under the curve (AUC, right). N = 6-11. *$p < 0.05$, **$p < 0.01$, ***$p < 0.005$, as compared with non-transgenic mice (Student's t-test).

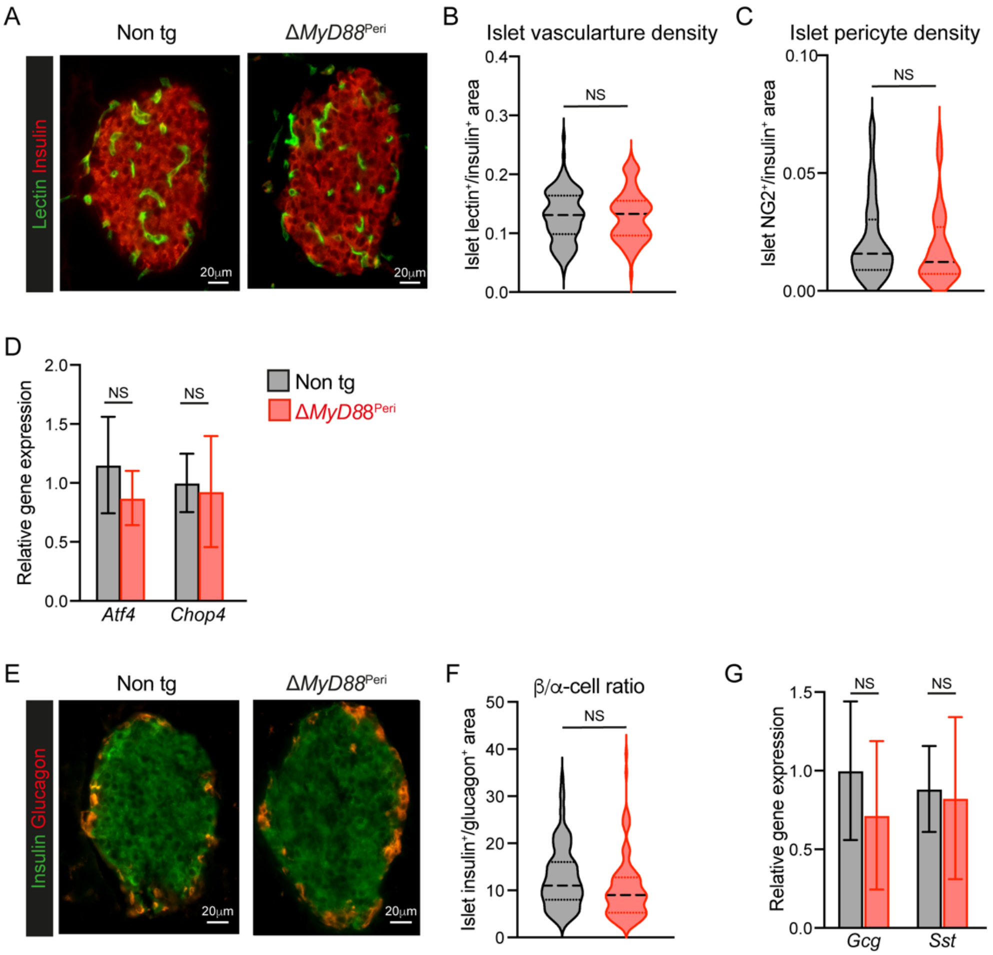

Supplemental Figure 4: Myd88-deficiency does not affect islet morphology and vasculature density

A, B) Analysis of the distribution of functional vasculature. Δ*MyD88*^{Peri} and non-transgenic mice were intravenously injected with tomato lectin (red) to label functional vessels. Pancreata were harvested from treated mice, and tissue sections were stained for insulin (blue bar). Images show the functional vasculature of representative islets (A), and violin diagrams show quantification of intra-islet vascular density (B). For each islet, the portion of the lectin$^+$ area out of the insulin$^+$ area was calculated. n > 100. NS, not significant (Student's t test).

C) Analysis of pericytes intra-islet density. Pancreatic tissues were stained for NG2 to label pericytes and insulin to label β-cells. The violin diagram shows a morphometric quantification of intra-islet pericyte density when the portion of NG2$^+$ area out of insulin$^+$ area was calculated per each islet. n > 70. NS, not significant (Student's t test).

D, **G**) Bar diagrams (mean ±SD) show β-cell gene expression analyzed by qPCR. Average levels in control islets were set to '1'. N = 5–8. NS, not significant (Student's t test).

E, F) Pancreatic tissues of non-transgenic (non-tg; left) and Δ*MyD88*^{Peri} (right) adult mice were immunostained for insulin (green) to label β-cells and glucagon (red) to label α-cells. Images (E) show representative islets, and violin diagrams (F) show the ratio between α - and β-cells in each islet. n > 130. NS, not significant (Student's t test).

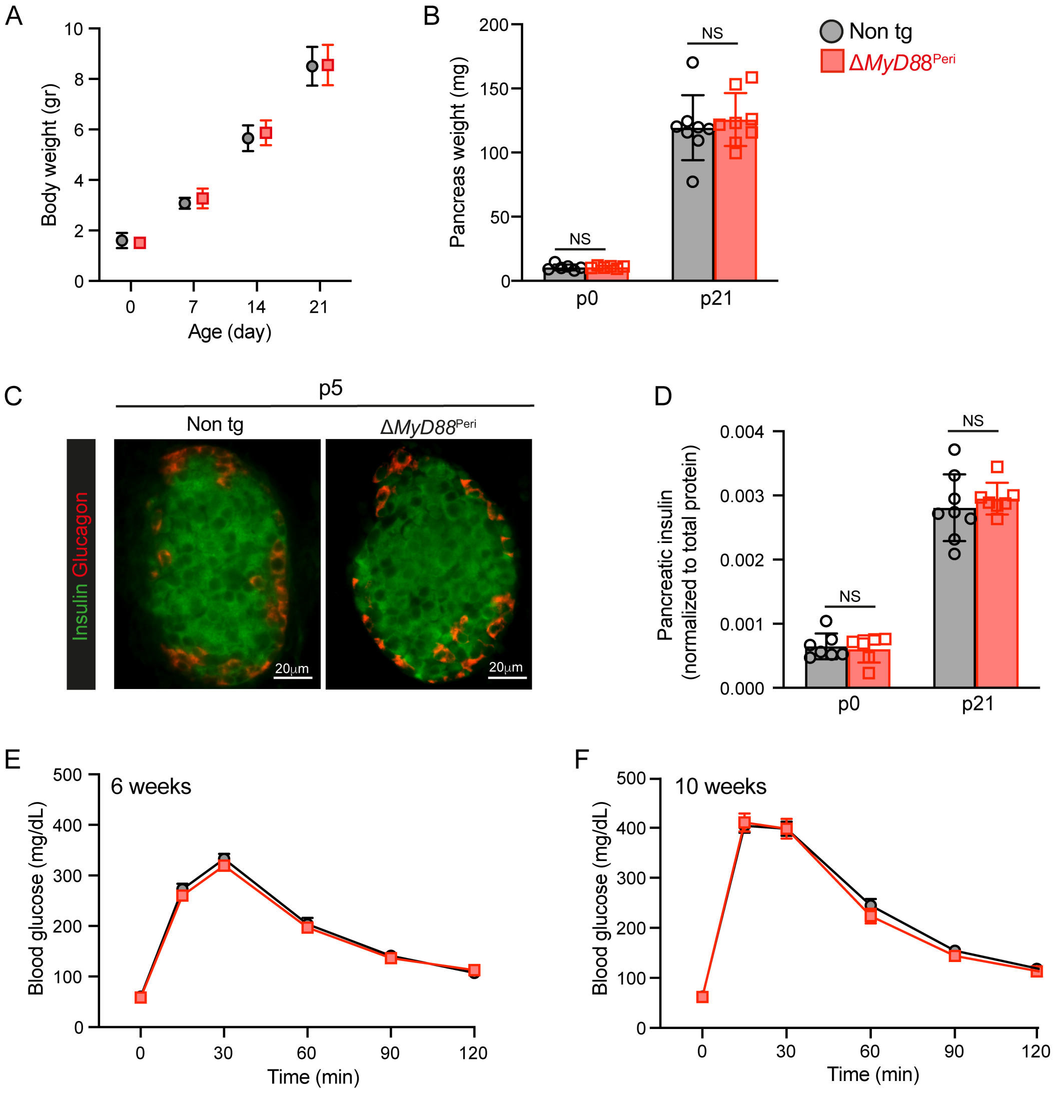

Supplemental Figure 5: Neonatal insulin production and pre-adult glucose tolerance are maintained in Δ*MyD88*^{Peri} mice

Δ*MyD88*^{Peri} (red) and non-transgenic littermates (Cre-negative; "non tg"; gray) mice were analyzed.
A) Pups were weighed at the indicated ages. Shown are mean (± SEM) body weight. N=6-8.
B) Bar diagrams (mean ±SD) show pancreatic weight at postnatal days (p) 0 and 21 pups. N=6-8. Each dot represents a single mouse. NS, not significant (Student's t test).
C) Pancreatic tissues of non-transgenic (non-tg; left) and Δ*MyD88*^{Peri} (right) mice at p5 were immunostained for insulin (green) and glucagon (red). Representative islets are shown.
D) Bar diagram (mean ±SD) shows pancreatic insulin content at p0 and p21, normalized to protein content. N = 6-8. Each dot represents a single mouse. NS, not significant (Student's t test).
E, F) IPGTT analysis of six (E; N=5) and 10 (F; N=16) weeks-old male mice. Shown are mean (± SEM) blood glucose levels.

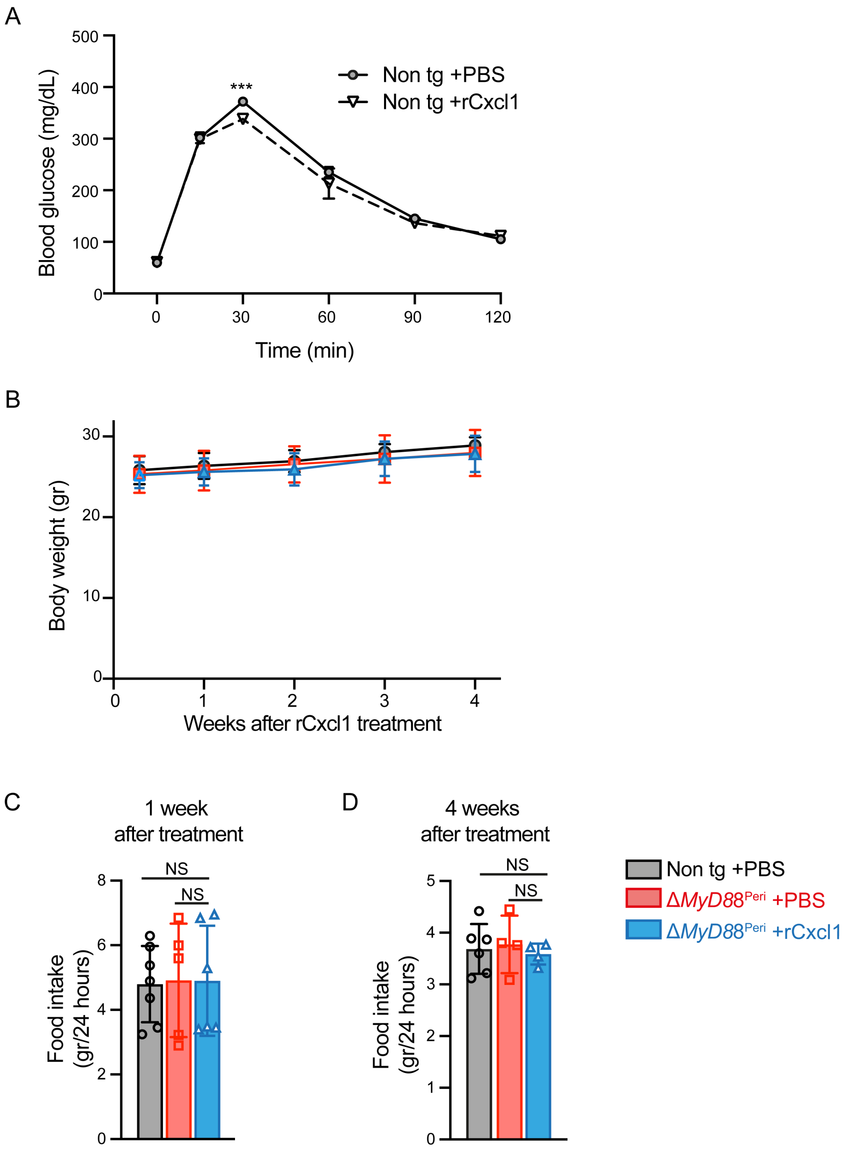

Supplemental Figure 6: Treatment with recombinant Cxcl1 does not affect weight gain and food intake

A) IPGTT of non-transgenic mice treated with recombinant mouse Cxcl1 (rCxcl1; dashed line) or PBS (solid line) a week before analysis. Shown are mean (± SEM) blood glucose levels. N = 4-8. ***p < 0.005 (Student's t test).
B) Δ*MyD88*^{Peri} male mice were treated with rCxcl1 (blue) at 14 weeks. Shown are weekly measurements of body weight (mean ±SD) following treatment compared to age- and sex-matched untreated non-transgenic (gray) and Δ*MyD88*^{Peri} (red) control mice. N=4-8.
C, **D**) 14-week-old Δ*MyD88*^{Peri} male mice were treated with rCxcl1 (blue) or PBS (red), and non-transgenic (gray) male mice were treated with PBS. Shown is the weight (mean ±SD) of food consumed over a 24-hour period by a single mouse, one (C) and four (D) weeks after rCxcl1 treatment. N=4-7. Each dot represents a single mouse. NS, not significant (ANOVA with Tukey's post-hoc).

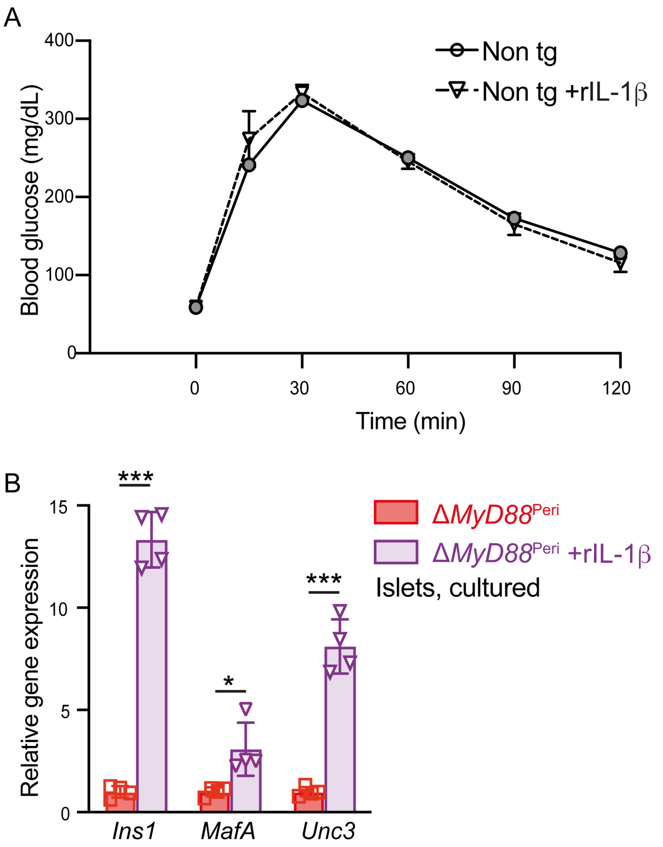

Supplemental Figure 7: Recombinant IL-1β promotes β-cell gene expression in Δ*MyD88*^{Peri} islets *in vitro*

A) IPGTT of non-transgenic 15-week-old mice treated with recombinant mouse IL-1β (dashed line) or PBS (solid line) a day before analysis. Shown are mean (± SEM) blood glucose levels. N = 4-5.

B) qPCR analysis of islets isolated from untreated Δ*MyD88*^{Peri} mice cultured for 24 hours in a medium either containing IL-1β (purple) or not (red). N= 4. *$p < 0.05$, ***$p < 0.005$; (Student's t test) compared to untreated mice. Each dot represents a single sample.

Supplemental Table 3. List of primary antibodies

Antigen	Antibody	Source	Identifier	Application
CD11b	Rat anti-CD11b, APC-conjugated (clone M1/70)	BioLegend	Cat# 101211, RRID:AB_312794	Flow cytometry
CD11c	Armenian Hamster anti-CD11c, APC-conjugated (clone N418)	Thermo Fisher Scientific	Cat# 17-0114-81, RRID:AB_469345	Flow cytometry
	Armenian Hamster anti-CD11c, PE-conjugated (clone N418)	BioLegend	Cat# 117307, RRID:AB_313776	Flow cytometry
CD115	Rat anti-CD115 (CSF-1R), Alexa Fluor 488-conjugated (clone AFS98)	BioLegend	Cat# 135512, RRID:AB_11218983	Flow cytometry
CD16 /CD32 (FcR)	Rat anti-CD16/CD32 (clone 93)	Thermo Fisher Scientific	Cat# 14-0161-85, RRID:AB_467134	Flow cytometry
CD19	Rat anti-CD19, PE-conjugated (clone 1D3/CD19)	BioLegend	Cat# 152407, RRID:AB_2629816	Flow cytometry
CD3	Rat anti-CD3, APC-conjugated (clone 17A2)	BioLegend	Cat# 100235, RRID:AB_2561455	Flow cytometry
CD31	Rat anti-CD31 (PECAM1), PE-conjugated (clone 390)	BioLegend	Cat# 102407, RRID:AB_312902	Flow cytometry
	Rat anti-CD31(PECAM1) (clone MEC13.3)	BD Bioscience	Cat# 553370, RRID:AB_394816	Immunofluorescence Primary
CD45	Rat anti-CD45, APC-conjugated (clone 30-F11)	Thermo Fisher Scientific	Cat# 17-0451-82, RRID:AB_469392	Flow cytometry
	Rat anti-CD45, FITC-conjugated (clone S18009F)	BioLegend	Cat# 157213, RRID:AB_2894427	Flow cytometry
	Rat anti-CD45, PE-conjugated (clone 30-F11)	BioLegend	Cat# 103106, RRID:AB_312971	Flow cytometry
	Rat anti-CD45, PerCP-conjugated (clone 30-F11)	BD Biosciences	Cat# 557235, RRID:AB_396609	Flow cytometry
CD64	Mouse anti-CD64 (FcγRI), FITC-conjugated (clone X54-5/7.1)	BioLegend	Cat# 139316, RRID:AB_2566556	Flow cytometry
	Mouse anti-CD64 (FcγRI), PE-conjugated (clone X54-5/7.1)	BioLegend	Cat# 139304, RRID:AB_10612740	Flow cytometry
Glucagon	Rabbit anti-glucagon (clone 13D11.33)	Millipore	Cat# AB932, RRID:AB_2107329	Immunofluorescence
Iba1	Rabbit anti-Iba1 (polyclonal)	FUJIFILM Wako Chemicals	Cat# 01919741, RRID: AB_839504	Immunofluorescence
Insulin	Guinea pig anti-insulin (polyclonal)	Agilent	Cat# IR002, RRID:AB_2800361	Immunofluorescence
NG2	Rabbit anti-NG2 (clone 132.38)	Millipore	Cat# AB5320, RRID:AB_91789	Immunofluorescence
TLR4	Mouse anti-TLR4 (clone1203B)	R&D systems	Cat# MAB27591, RRID: AB_2271967	Immunofluorescence

Supplemental Table 4. List of primers and probes for qPCR

**Primers**	**Method**	**Sequence**
Abcc8 (Sur1)	Taqman	Mm00803450_m1
Atf4	Taqman	Mm00515324_m1
Cyclophilin	SYBR green	TGCCGCCAGTGCCATT<br>TCACAGAATTATTCCAGGATTC
Cxcl1	Taqman	Mm04207460_m1
Ddit3 (Chop)	Taqman	Mm00492097_m1
GAPDH	Taqman	TGCACCACCAACTGCTTAG<br>GGATGCAGGGATGATGTTC<br>Probe: CAGAAGACTGTGGATGGCCCCTC
Gcg	Taqman	Mm01269055_m1
Igf1	Taqman	Mm00439560_m1
Il1b	Taqman	Mm00434228_m1
Il1rl1	Taqman	Mm00516117_m1
Ins1	SYBR green	GGGTCGAGGTGGGCC<br>CTGCTGGCCTCGCTTGC
Ins2	SYBR green	GGCTGCGTAGTGGTGGGTCTA<br>CCTGCTCGCCCTGCTCTT
Kcnj11 (Kir6.2)	SYBR green	GGACCTCCGAAAGAGCATGA<br>GCGCACCACCTGCATGT
MafA	SYBR green	GCTGGTATCCATGTCCGTGC<br>TGTTTCAGTCGGATGACCTCC
MyD88	Taqman	Mm00440338_m1
NeuroD1	SYBR green	ATGACCAAATCATACAGCGAGAG<br>TCTGCCTCGTGTTCCTCGT
Nkx6-1	SYBR green	TCAGGTCAAGGTCTGGTTCCA<br>CGGTCTCCGAGTCCTGCTT
Pdx1	SYBR green	CCCCAGTTTACAAGCTCGCT<br>CTCGGTTCCATTCGGGAAAGG
Slc2a2 (Glut2)	SYBR green	TCAGAAGACAAGATCACCGGA<br>GCTGGTGTGACTGTAAGTGGG
Sst	Taqman	Mm00436671_m1
Tlr4	Taqman	Mm00445273_m1
Unc3	Taqman	Mm00453206_s1

Supplemental Table 5. List of secondary antibodies

Antibody	Source	Identifier
Donkey Alexa Fluor 488-labeled anti-goat IgG (polyclonal)	Thermo Fisher Scientific	Cat# A-11055, RRID:AB_2534102
Donkey Alexa Fluor 555-labeled anti-rabbit IgG (polyclonal)	Thermo Fisher Scientific	Cat# A-31572, RRID:AB_162543
Donkey Alexa Fluor 555-labeled anti-rat IgG (polyclonal)	Abcam	Cat# ab150154, RRID:AB_2813834
Goat Alexa Fluor 488-labeled anti-guinea pig IgG (polyclonal)	Thermo Fisher Scientific	Cat# A-11073, RRID:AB_2534117
Goat Alexa Fluor 488-labeled anti-rabbit IgG (polyclonal)	Thermo Fisher Scientific	Cat# A-11034, RRID:AB_2576217
Goat Alexa Fluor 555-labeled anti-guinea pig IgG (polyclonal)	Thermo Fisher Scientific	Cat# A-21435, RRID:AB_2535856

References

1. Elgamal RM, et al. An Integrated Map of Cell Type–Specific Gene Expression in Pancreatic Islets. *Diabetes*. 2023;72(11):1719–1728.

2. Sakhneny L, et al. Pancreatic Pericytes Support β-Cell Function in a Tcf7l2-Dependent Manner. *Diabetes*. 2018;67(3):437–447.
